# Brain and Serum Androsterone Is Elevated in Response to Stress in Rats with Mild Traumatic Brain Injury

**DOI:** 10.3389/fnins.2016.00379

**Published:** 2016-08-26

**Authors:** Richard J. Servatius, Christine E. Marx, Swamini Sinha, Pelin Avcu, Jason D. Kilts, Jennifer C. Naylor, Kevin C. H. Pang

**Affiliations:** ^1^Department of Veterans Affairs, Syracuse Veterans Affairs Medical CenterSyracuse, NY, USA; ^2^Rutgers Biomedical Health Sciences, Stress and Motivated Behavior Institute, Rutgers UniversityNewark, NJ, USA; ^3^Graduate School of Biomedical Sciences, Rutgers UniversityNewark, NJ, USA; ^4^Veterans Affairs Mid-Atlantic Mental Illness, Research Education and Clinical Center, Durham Veterans Affairs Medical CenterDurham, NC, USA; ^5^Department of Psychiatry and Behavioral Sciences, Duke University School of MedicineDurham, NC, USA; ^6^Department of Veterans Affairs, New Jersey Health Care SystemEast Orange, NJ, USA

**Keywords:** acoustic startle response, lateral fluid percussion, corticosterone, concussions, Sprague-Dawley, allopregnanolone, pregnenolone, footshock

## Abstract

Exposure to lateral fluid percussion (LFP) injury consistent with mild traumatic brain injury (mTBI) persistently attenuates acoustic startle responses (ASRs) in rats. Here, we examined whether the experience of head trauma affects stress reactivity. Male Sprague-Dawley rats were matched for ASRs and randomly assigned to receive mTBI through LFP or experience a sham surgery (SHAM). ASRs were measured post injury days (PIDs) 1, 3, 7, 14, 21, and 28. To assess neurosteroids, rats received a single 2.0 mA, 0.5 s foot shock on PID 34 (S34), PID 35 (S35), on both days (2S), or the experimental context (CON). Levels of the neurosteroids pregnenolone (PREG), allopregnanolone (ALLO), and androsterone (ANDRO) were determined for the prefrontal cortex, hippocampus, and cerebellum. For 2S rats, repeated blood samples were obtained at 15, 30, and 60 min post-stressor for determination of corticosterone (CORT) levels after stress or context on PID 34. Similar to earlier work, ASRs were severely attenuated in mTBI rats without remission for 28 days after injury. No differences were observed between mTBI and SHAM rats in basal CORT, peak CORT levels or its recovery. In serum and brain, ANDRO levels were the most stress-sensitive. Stress-induced ANDRO elevations were greater than those in mTBI rats. As a positive allosteric modulator of gamma-aminobutyric acid (GABA_A_) receptors, increased brain ANDRO levels are expected to be anxiolytic. The impact of brain ANDRO elevations in the aftermath of mTBI on coping warrants further elaboration.

## Introduction

Diffuse mild traumatic brain injury (mTBI), as induced through lateral fluid percussion (LFP), affects the brain in a generally accepted cascade with structural (Povlishock et al., 1979, 1983), metabolic (Frey et al., 2014), neurochemical (Zhong et al., 2005), neuroimmune (Redell et al., 2013) adjustments. Although dramatic shifts are apparent in the hours and days following mTBI, inflammatory processes and activation of astrocytes and glial extend weeks to months after injury. Emotional dysfunction may become manifest as depression or anxiety (Hoge et al., 2008; Matthews et al., 2011; Wilk et al., 2012; Bryan et al., 2013), reflecting a reduced ability to cope with stress (Bohnen et al., 1992; Snell et al., 2011).

Evidence is accumulating that neurosteroids participate in emotional regulation (Sripada et al., 2013a,b, 2014) and are potential targets as therapeutics in mood and anxiety disorders (Pinna and Rasmusson, 2012; Brown et al., 2014; Marx et al., 2014; Pinna, 2014). Neurosteroids as a class of neuromodulators are fairly complex and regionally distributed within the brain (Morrow, 2007). Depending upon the specific neurosteroid, they enhance or inhibit ligand gated γ-aminobutyric acid (GABA_A_) receptor responses; a number of neurosteroids also act at glutamate N-methyl-D-aspartate (NMDA) receptors, among others. PREG is a parent compound which can produce a number of active neurosteroids through metabolizing and enzymatic steps. PREG allosterically potentiates NMDA receptors, while negatively modulating GABA_A_ receptors (Mellon et al., 2001). In contrast, ALLO and ANDRO, which are downstream, are potent positive allosteric GABA_A_receptor modulators (Wilson and Biscardi, 1997) that have anxiolytic and antidepressant effects (Marx et al., 2006a; Girdler and Klatzkin, 2007; Schule et al., 2011, 2014; Ben Dor et al., 2015). PREG has been found to be lower in the cerebrospinal fluid of unmedicated, clinically depressed patients (George et al., 1994). Lower ALLO levels were found in the cerebrospinal fluid of women with PTSD (Rasmusson et al., 2006). Together, these observations suggest patterns of neurosteroids are important for understanding stress-related mental health difficulties.

Neurosteroids also demonstrate multiple actions that are relevant to the pathophysiology of mTBI: neuroprotection, anti-inflammatory, and neurotrophic effects (Djebaili et al., 2005; Vanlandingham et al., 2007, 2008; Sayeed et al., 2009). Neurosteroids also alter the course of pathology after TBI (Stein, 2011) and mTBI (Milman et al., 2008). It is not known to the degree acute stressors affect neurosteroids during the course of recovery from mTBI.

Studies of peripheral stress-responsive steroids in the aftermath of mTBI are equivocal. Blunted CORT responses were observed in rats exposed to controlled cortical injury 4 weeks after injury (Taylor et al., 2006, 2013). Exaggerated CORT responses were also apparent after a similar injury and in similar time frames (Taylor et al., 2008). In response to LFP injury, this group reported exaggerated responses (Griesbach et al., 2011)—but only after peak levels were divided by basal levels which were lower in injured rats. Raw CORT values indicated blunted responses 7 and even 14 days after injury. Together, these studies suggest blunted CORT reactivity following TBI. However, the stress procedures involved extended exposure to aversive stimulation (e.g., forced exercise) and may therefore reflect adaptation to continued stress, not the stress response *per-se*.

The present study examined whether experience of mTBI alters stress reactivity to moderately stressful discrete experiences compared to sham surgery. Simple stress reactivity was assessed in terms of plasma CORT levels and recovery in response to a single foot shock. Neurosteroids were assessed in serum and brain regions (hippocampus, prefrontal cortex, and cerebellum) of rats exposed to either a single foot shock (simple reactivity) or two daily foot shocks (sensitization to homotypic stressors). Moreover, ASRs were repeatedly measured as a tracking biomarker of mTBI (Pang et al., 2015). It is presumed that persistently attenuated ASRs reflect neuroinflammation (Lu et al., 2003; Lu and Moochhala, 2004). Against this background, we expected suppressed CORT responses, blunted simple neurosteroid reactivity, but sensitized neurosteroid responses.

## Methods

### Husbandry

Adult (aged 12–14 weeks) male Sprague-Dawley rats were obtained from Charles River Laboratories (Kingston, NY). Upon arrival, rats, were single-housed and maintained on a 12:12h light:dark cycle (0700 lights on) with access to laboratory chow and water *ad libitum*. Rats were acclimated to the housing conditions for 2 weeks prior to experimentation. Temperatures of housing and preparation rooms were maintained between 22 and 25°C. All experimental procedures occurred between 0700 and 1100 h. The study was approved by the Department of Veterans Affairs New Jersey Health Care System Institutional Animal Care and Use Committee in accordance with AAALAC standards.

### Experimental design

**Figure 1:**
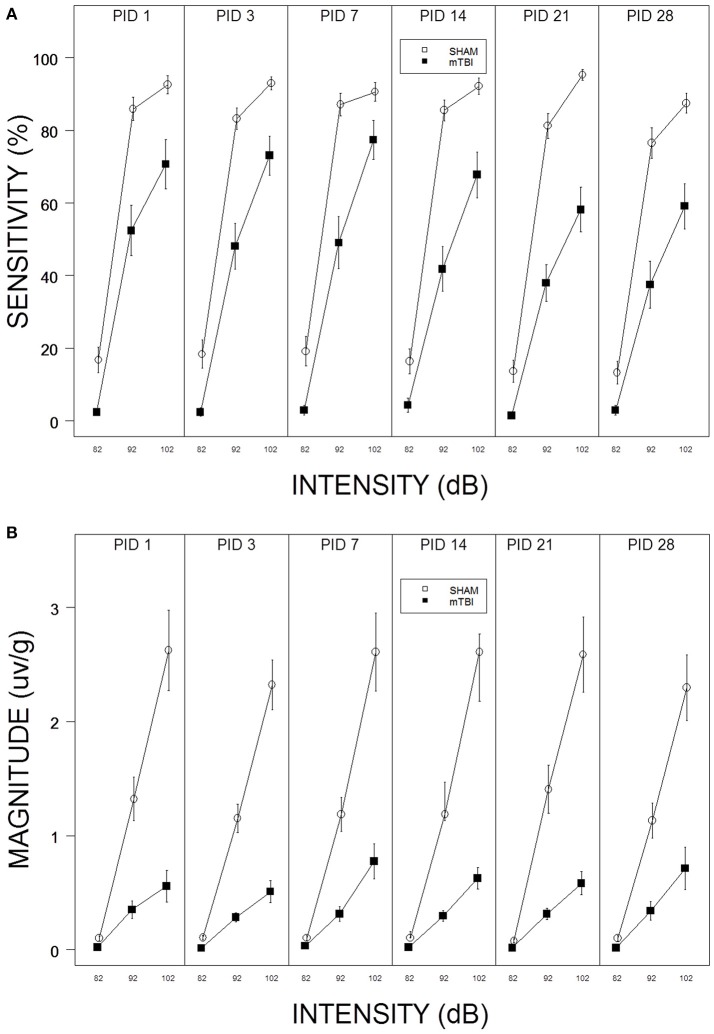
**(A)** Repeated testing of ASR sensitivity in mTBI and SHAM rats. Each panel depicts an ASR test session. The legend is contained with the figure. mTBI rats exhibited attenuated sensitivity from PID 1 through PID 28. There was no evidence of recovery; sensitivity of mTBI rats decreased from PID 1 to PID 28. **(B)** Repeated testing of ASR magnitude in mTBI and SHAM rats. Each panel depicts an ASR test session. The legend is contained with the figure. mTBI rats exhibited attenuated ASR magnitudes from PID 1 through PID 28. There was no evidence of recovery.

An initial ASR test was performed. Rats were matched for amplitudes to the 102 dB stimulus, and then randomly assigned to SHAM and mTBI conditions. The time line of stress, behavioral tests, and sample collection are all relative to the day of injury as post-injury day (PID) and are depicted in Figure 1. The ASR was tested PIDs 1, 3, 7, 14, 21, and 28. Within the SHAM and mTBI groups rats were randomly assigned to one of the following conditions: exposure to the conditioning context chamber (CON group) without shock on PID 34 (SHAM, *N* = 7; mTBI, *N* = 7), exposure to a single shock on PID 34 (S34 group; SHAM, *N* = 8; mTBI, *N* = 7), exposure to the conditioning chamber on PID 34 and a single shock on PID 35 (S35 group; SHAM, *N* = 8; mTBI, *N* = 6), or exposure to a single shock on both PID 34 and PID 35 (2S group; SHAM, *N* = 8; mTBI, *N* = 6). To characterize the stress response and its recovery, the two-shock group (SHAM and mTBI) had a basal blood sample at 0700. At 0900, the these groups received a single shock and were returned to their home cages with blood samples obtained through tail nick at 15, 30, and 60 min after shock. For all groups, a terminal sample was obtained 15 min after chamber exposure either on PID 34 or on PID 35.

### Fluid percussion injury

Fluid percussion injury was induced as previously described (Pang et al., 2015). All rats underwent surgery (−3.0 mm AP, −3.5 mm ML from Bregma; 4 mm diameter) to expose an intact dural surface. Anesthesia for surgery was a mixture of ketamine (60 mg) and xylazine (7 mg) delivered i.p. at 1.0 ml/kg. A leur-lok connector, supported by a section of 12 ml syringe, was inserted into the skull and fixed with dental cement and metal screws. The location of the craniotomy was counterbalanced between right and left hemispheres. The following day, rats were anesthetized with 5% isoflurane (about 60–90 s). A voice-coil controlled device (Wahab et al., 2015) was attached through tubing to the leur-lok connector. Injury was induced when the rat exhibited bilateral foot-pinch reflexes. A single pressure pulse (20.0 ± 0.77 psi peak pressure for mTBI, 0.0 psi for SHAM) was delivered by the device to the cranial surface. Immediately upon delivery of the pressure pulse, apnea (latency to first observable inspiration) and the appearance of tail/limb hyper-extension were observed, then rats were placed supine in an observation cage and righting reflex (latency to righting to prone position on all four limbs) was observed. The injury parameters and immediate responses to injury are contained in Table 1. The degree LFP injury affected apnea, righting reflex, and hyperextension is similar to that of earlier work (Pang et al., 2015).

**Table 1:** **Parameters and immediate outcomes of exposure to lateral fluid percussion injury in rats**.

	**Peak pressure (psi)**	**Apnea (s)**	**Righting reflex (s)**	**Hyperextension**
SHAM	0	none	77 ± 17.8	0
mTBI	20 ± 0.77	12.9 ± 1.7	474 ± 31.6	26

### Stress

The apparatus was described previously (Servatius et al., 2008). Foot-shock exposure occurred in an operant chambers (Coulbourn Instruments, Langhorn, PA), enclosed in a sound attenuated box (Coulbourn Instruments, Langhorn, PA), equipped with constant current shockers (Coulbourn Instruments, Langhorn, PA). Graphic State Notation software (v. 3.02, Coulbourn Instruments, Langhorn, PA) controlled the foot-shock delivery. A single 2.0 mA foot-shock (0.5 s duration) was delivered through the grid floor (Coulbourn Instruments, Langhorn, PA) after 1 min in the chamber. Context controls (CON) rats were transferred to the chamber and remained in the chamber for 1 min before transfer back to the home cage.

### Behavioral tests

The acoustic startle reflex (ASR) procedure was conducted as previously described (Servatius et al., 1998; Pang et al., 2015). Rats were allowed to acclimate to the testing apparatus for 10 min prior to the onset of testing. Each test session consisted of 24 presentations of white noise of either 82, 92, or 102 dB (100 ms with 5 ms rise/fall) delivered against a continuous background noise level of 68 dB. Stimuli were delivered in a single pseudorandom order which had the constraint the one each of the three stimulus intensity would be delivered in three consecutive trials with interstimulus intervals of 25–35 s. As in previous work (Pang et al., 2015), a startle response was defined as platform movement within 250 ms after stimulus presentation that significantly exceeded 250 ms baseline movements. The protocol (Servatius et al., 1998; Pang et al., 2015) yields measures of sensitivity (% responses) and responsivity (magnitude of responses; Blumenthal and Goode, 1991) for the three stimulus intensities.

### Tissue collection

To assess stress reactivity, blood samples were obtained via tail nick as previously described (Servatius et al., 1994). Briefly, blood samples were obtained from a lateral tail vein into heparinized (EDTA contained) capillary tubes (100 ml) in duplicate while the rats were loosely restrained on a sampling table. Individual sampling required <30 s from the time of removal from the home cage to the completion of the duplicate sample. Plasma was separated then stored in non-heparinized capillary tubes at −80°C until assayed for CORT through radioimmunoassay (RIA; CORT I125 RIA kit, MP Biomedicals, Inc., Orangeburg, NY) according to the vendor's instructions. Sacrifice was by rapid decapitation without sedatives 15 min after receiving footshock or placement in experimental context. Trunk blood was collected into serum separator tubes and centrifuged at 2300 rpm for 15 min at 4°C and stored at −80°C until assayed. The brain was extracted and bilateral pre-frontal cortex, bilateral hippocampus and the cerebellum were fresh dissected and frozen at −80°C until assayed. Brain dissection required <30 s. The amount of brain tissue used for assay was 40–70 mg of prefrontal cortex, 60–70 mg for hippocampus, and 250–300 mg for cerebellum.

### Neurosteroids

Quantification was performed by highly sensitive and specific gas chromatography (GC)/mass spectrometry (MS), preceded by high performance liquid chromatography (HPLC) purification as previously described (Marx et al., 2006b,c; Sripada et al., 2013b, 2014), with some modifications. Serum (1.0 mL) was homogenized with 2.0 mL distilled water containing trace tritiated neurosteroid (New England Nuclear) to detect the HPLC fraction of interest, as well as a constant amount of deuterated allopregnanolone (D4-allopregnanolone) and deuterated pregnenolone (D4-pregnenolone) as the internal standards. Supernatants were extracted three times with three volumes of ethyl acetate. Brain tissues were initially suspended in 1.0 mL distilled water and extractions were performed with two volumes of ethyl acetate. HPLC purification was performed on an 1100 Series Agilent instrument. Standards and samples were derivatized utilizing heptafluorobutyric acid anhydride (HFBA) and injected onto an Agilent 5973 mass spectrometer (MS) coupled to an Agilent 6890 N GC equipped with an Agilent HP-5MS 30 m × 0.250 mm × 0.25 μm capillary column. Positive ion electron impact (EI) ionization was utilized in the GC/MS component using helium as the carrier gas. A subset of serum and brain samples were analyzed in duplicate in positive ion EI mode. In addition to the GC/MS retention time characteristic of each neurosteroid, the definitive structural identification of each neurosteroid was provided by its unique mass fragmentation pattern. Mass spectrometer single ion monitoring was used to focus on the most abundant ion fragment for each HFBA derivative [PREG (298.2), ALLO (496.2), and ANDRO (486.2)]. Internal standards consisted of deuterated D4-allopregnanolone for ALLO and ANDRO quantifications, and D4-pregnenolone for PREG quantification (Cambridge Isotope Laboratories, Andover, MA). For neurosteroid quantification, the standard curve for the steroid of interest was prepared by combining known quantities of the neurosteroid (Steraloids, Newport, RI) with a constant amount of deuterated internal standard. Identical to the samples, the standard curve was extracted three times in ethyl acetate prior to HPLC purification and GC/MS injection (standard curve *r*^2^ = 0.99 for each neurosteroid). The area under the curve of each known quantity of neurosteroid was divided by the area under the peak of the internal standard. This ratio was then plotted on the *y*-axis against known quantities of each steroid to generate the standard curve. Only peaks with a signal to noise ratio ≥5:1 were integrated. The limit of neurosteroid quantification with this methodology is 1 pg for PREG, ALLO, and ANDRO (femtomolar sensitivity). Intra-assay coefficients of variation were 2.2% for PREG, 3.4% for ALLO, and 4.7% for ANDRO. These 84 intra-assay coefficients of variation were in the same range as our prior investigations utilizing an EI approach.

### Data analysis

ASRs were computed as previously reported (Pang et al., 2015). The primary dependent measures are the proportion of responses by stimulus intensity (sensitivity) and the magnitude of responses by stimulus intensity (responsivity). Magnitude was displacement relative to the rat's body weight. These data were analyzed with ANOVA models with repeated measures for Stimulus Intensity and Sessions. Basal CORT levels were assessed separately from CORT stress responses, which were analyzed with a mixed ANOVA. For neurosteroids, multiple analyses of variance (MANOVAs) were computed. For serum, PREG, ALLO, and ANDRO were compared. In the brain, PREG, ALLO, and ANDRO were separately assessed by brain region (prefrontal cortex, hippocampus, and cerebellum). As the equivalent of an F statistic, Hotelling' Trace was used for the test of overall significance (*p* < 0.05), with Bonferroni *t*-tests used to understand *post-hoc* differences. All data are expressed as mean ± standard error of the mean.

## Results

### ASR

Sensitivity to respond decreased in the aftermath of mTBI and did not recover by PID 28. In fact, sensitivity of mTBI rats decreased from PID 14–28 (See Figure 1A). Thus, the suppressive effect of mTBI on ASR sensitivity is long lasting. These impressions were confirmed with a 2 × 3 × 6 (mTBI × Intensity × Session) mixed-ANOVA. The interaction of mTBI × Intensity × Session, *F*_(10, 560)_ = 2.2, *p* = 0.012, was significant and superseded all other main effects and interactions. Similarly, mTBI rats displayed ASR magnitudes that were suppressed and which did not change or recover (See Figure 1B). Only the main effects of Intensity, *F*_(2, 112)_ = 101.4, and TBI, *F*_(1, 56)_ = 39.5, were significant, *ps* < 0.001. The dramatic suppression evident in average responding extended to individuals rats in that only 3 of 26 mTBI rats recovered to 50% of their pre-injury ASR magnitude.

### CORT

**Figure 2:**
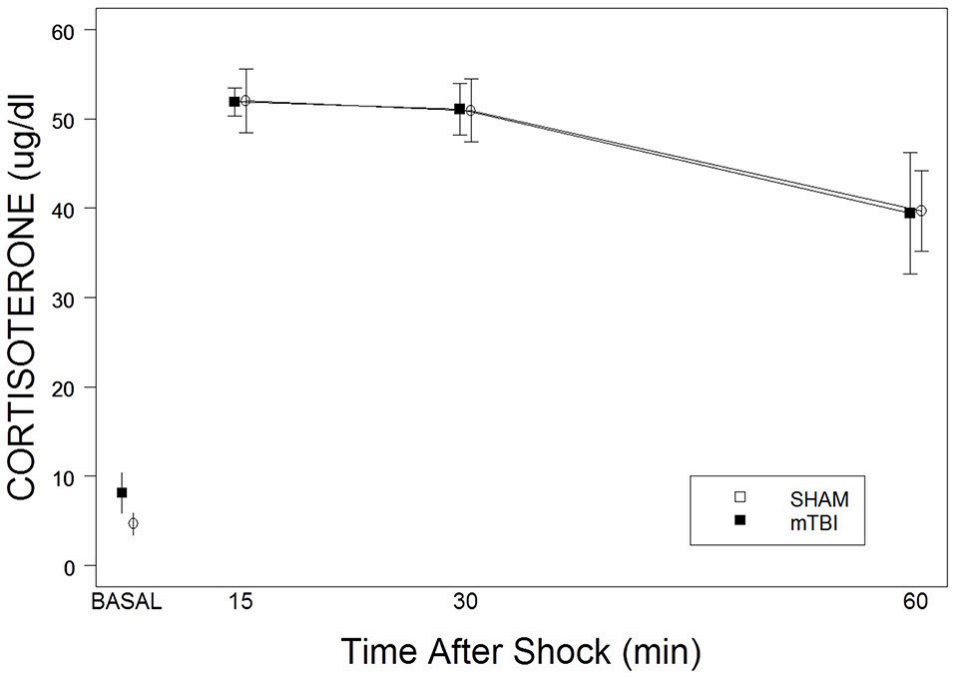
**Corticosterone basal levels and responses to a single 2.0 mA foot shock in mTBI and SHAM rats**. The legend is contained with the figure. No differences were detected in basal levels on PID 34. No differences were apparent in the magnitude of corticosterone levels or recovery over the period of 15–60 min after exposure to a single foot shock.

CORT levels, derived from basal samples obtained soon after lights on, did not differ between SHAM (4.6 ± 1.2 μg/dl) and mTBI (8.2 ± 2.3 μg/dl) rats, *t*_(8)_ = 1.3, *p* > 0.05. Repeated samples obtained after a single foot shock did not distinguish between SHAM and mTBI rats. This impression was confirmed with a 2 × 3 (TBI × Samples) mixed-ANOVA. Only the time factor, *F*_(2, 24)_ = 10.4, *p* < 0.01), was significant (See Figure 2). Essentially, SHAM and mTBI rats were isomorphic in stress response levels and recovery of CORT. Note that CORT levels from the single shock are consistent with those attributable to exposure to a moderately intense stressor.

### Neurosteroids

**Figure 3:**
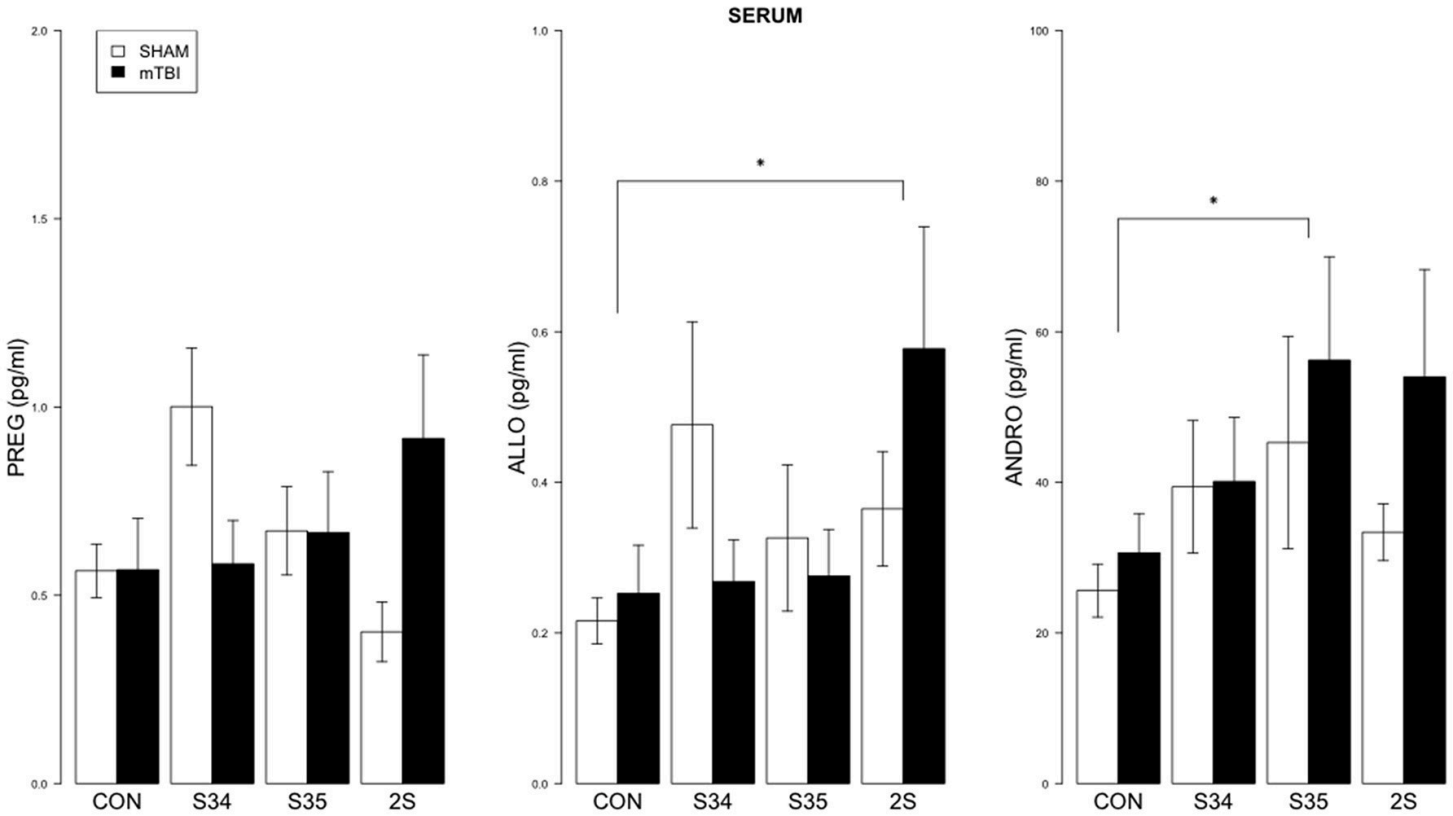
**Neurosteroid levels in serum as a function of stress exposure in mTBI and SHAM rats**. Serum levels of PREG, ALLO, and ANDRO are depicted in left, center, and right panels, respectively. The legend is contained within the left panel. For ALLO, 2S rats exhibited elevated levels relative to CON rats. For ANDRO, levels of S35 rats were elevated compared to CON rats. Asterisks indicate *p* < 0.05.

For serum, a 2 × 4 (mTBI × Stress Group) MANOVA indicated significant effect of Stress Group, *F*_(9, 137)_ = 1.99, *p* < 0.05 (See Figure 3). Significant comparisons were indicated for ALLO (CON vs. 2S) and ANDRO (CON vs. S35), all *ps* < 0.05.

**Figure 4:**
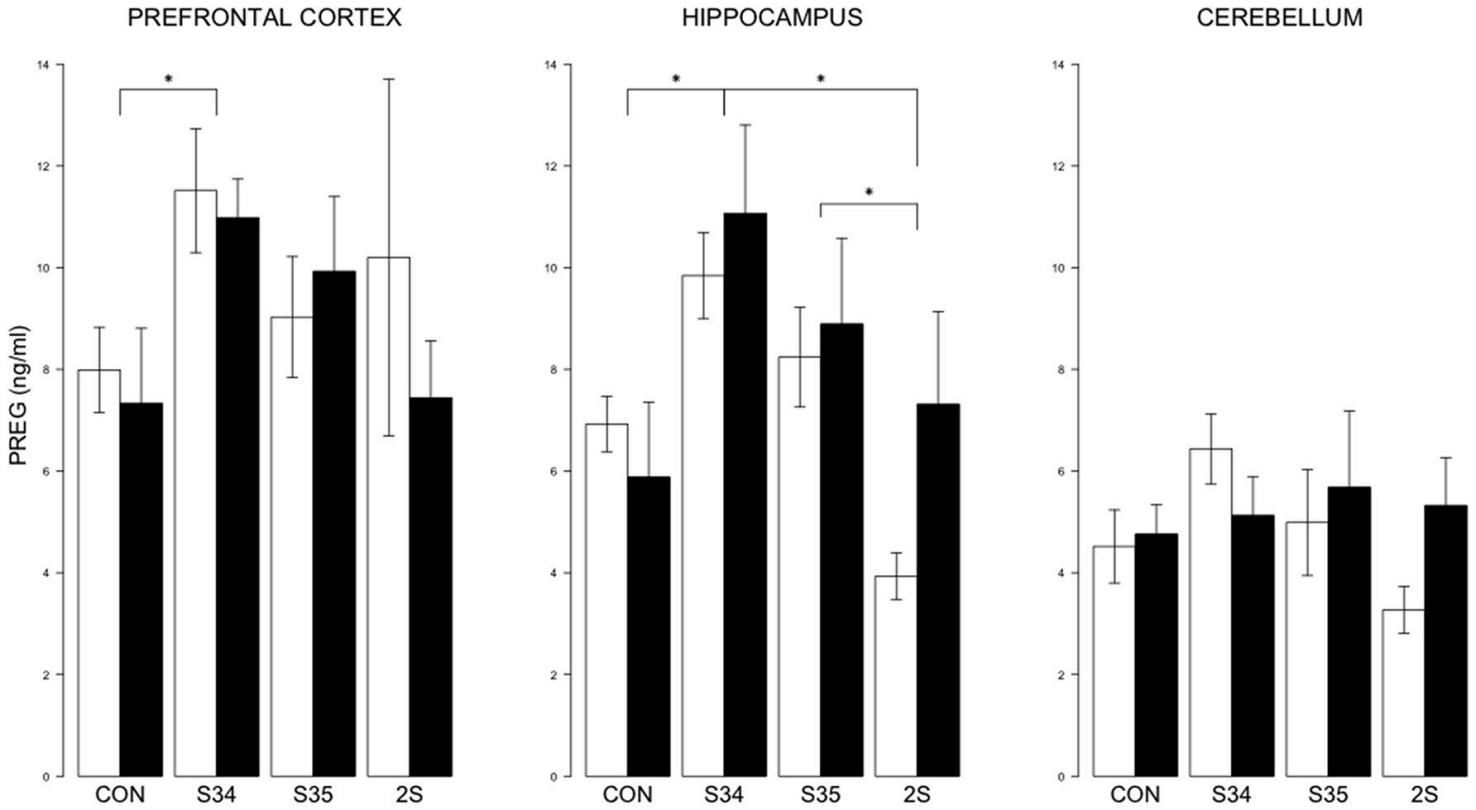
**PREG levels in the prefrontal cortex, hippocampus, and cerebellum as a function of stress in mTBI and SHAM rats**. The legend is same as Figure 3. In prefrontal cortex, PREG levels of S34 rats were greater than those of CON rats. In the hippocampus, PREG levels of S34 rats were greater than CON rats and 2S rats. Also, PREG levels of S35 rats were greater than 2S rats. Asterisks indicate *ps* < 0.05.

For brain PREG, a 2 × 4 (mTBI × Stress Group) MANOVA indicated an effect of Stress Group, *F*_(9, 131)_ = 0.013 (See Figure 4). In the prefrontal cortex, PREG levels were elevated in S34 rats compared to CON rats, *p* < 0.05. In the hippocampus, PREG levels were elevated in S34 rats compared to C and 2S rats, and in S35 rats compared to 2S rats, *ps* < 0.05. Further, in the hippocampus and cerebellum, PREG levels were elevated in S34 rats compared to 2S rats, *ps* < 0.05. In the cerebellum, PREG levels were elevated in S34 rats compared to 2S rats.

**Figure 5:**
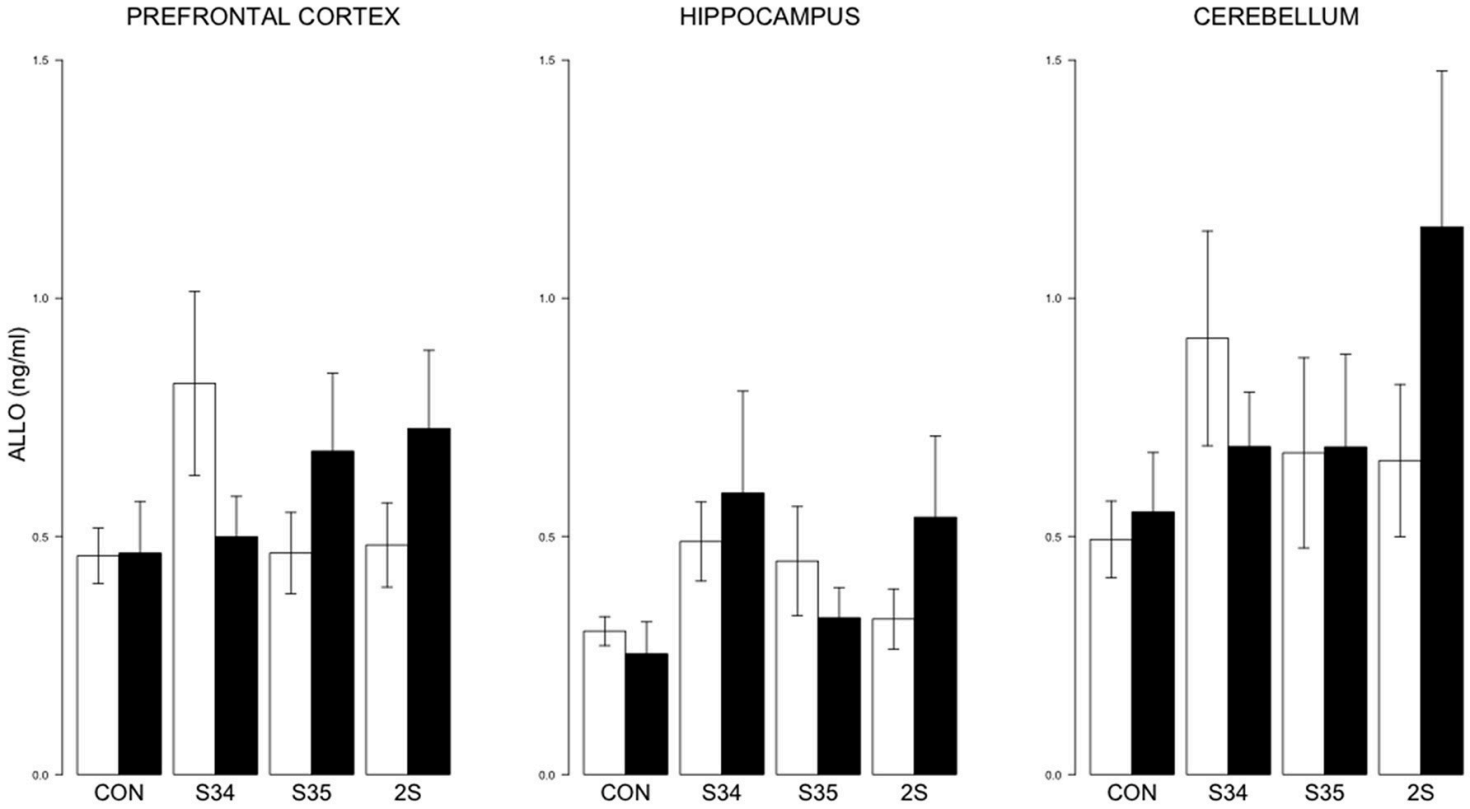
**ALLO levels in the prefrontal cortex, hippocampus, and cerebellum as a function of stress in mTBI and SHAM rats**. The legend is the same as Figure 3. No significant differences were indicated.

For brain ALLO, no differences were detected (See Figure 5).

**Figure 6:**
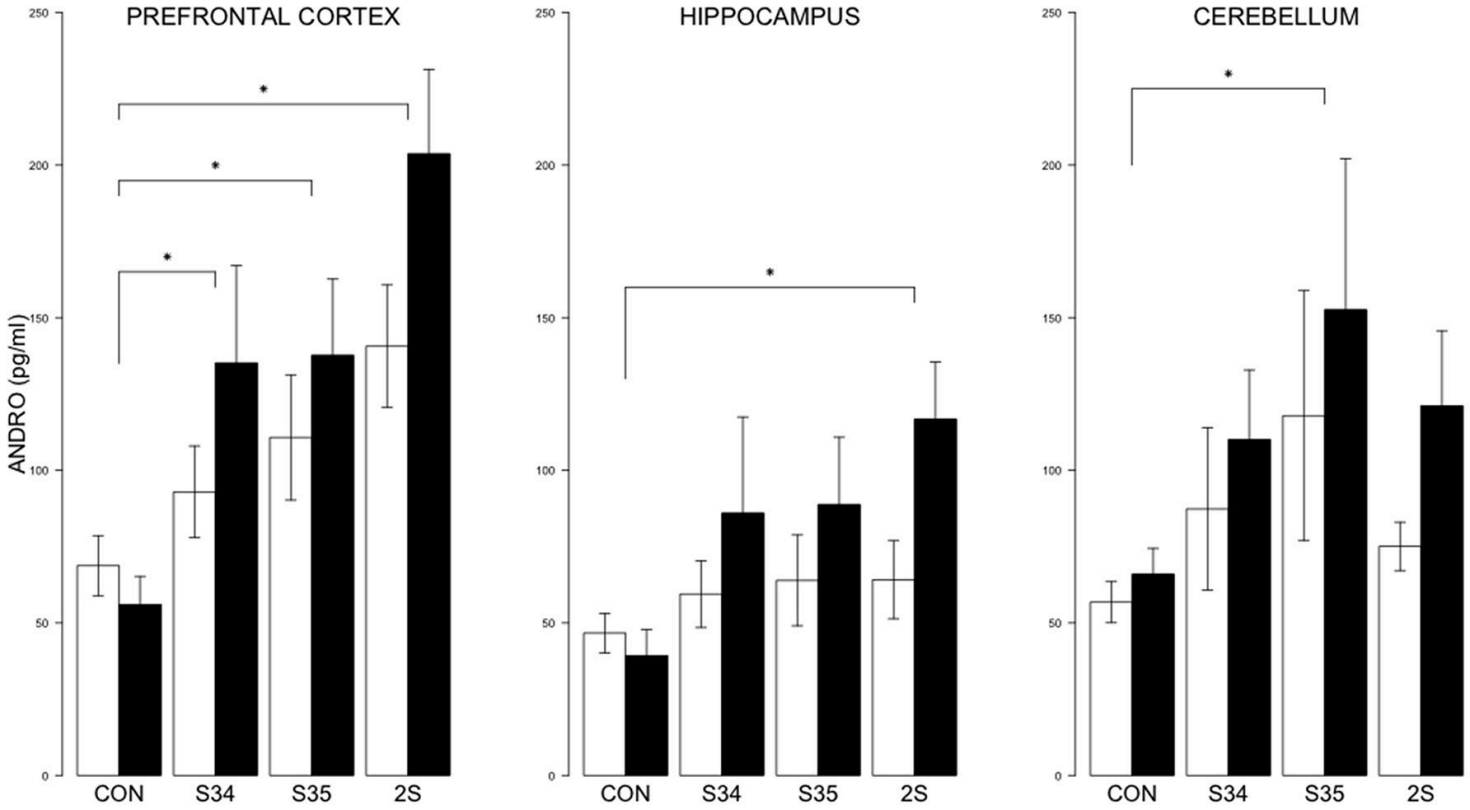
**ANDRO levels in the prefrontal cortex, hippocampus, and cerebellum as a function of stress in mTBI and SHAM rats**. The legend is the same as Figure 3. In prefrontal cortex, ANDRO levels of the S34, S35, and 2S groups were higher than those of CON rats. In the hippocampus, ANDRO levels of 2S rats were greater than those of CON rats. In the cerebellum, ANDRO levels of s35 rats were greater than CON rats. ANDRO levels of mTBI rats were greater than SHAM rats in prefrontal cortex and hippocampus. Asterisks indicate *ps* < 0.05.

For brain ANDRO, the 2 × 4 (mTBI × Stress Group) MANOVA indicated significant effects of Stress Group, *F*_(9, 144)_ = 5.8, and mTBI, *F*_(3, 46)_ = 3.4, *ps* < 0.05 (See Figure 6). In the prefrontal cortex, the levels of ANDRO of S34, S35 and 2S rats were all elevated compared to CON rats; ANDRO levels of 2S rats were elevated compared to S34 and S35 rats which did not differ, all *ps* < 0.05. In the hippocampus, ANDRO levels of 2S rats were elevated compared to CON rats, *p* < 0.05. In the cerebellum, S35 ANDRO levels were elevated compared to CON rats. The ANDRO levels of mTBI rats were higher than those of CON rats in the prefrontal cortex and hippocampus, *ps* < 0.05. Inasmuch as ANDRO levels of mTBI-CON rats were nominally lower than SHAM-CON rats in both brain regions, elevations are solely from the experience of stress.

Specific *a priori* comparisons were separately evaluated: (1) whether TBI induced changes in brain neurosteroids in the absence of stress and (2) whether sensitization was apparent and to a greater degree in mTBI rats. MANOVAs of these two specific comparisons were nonsignificant.

## Discussion

In the aftermath of mTBI, the subacute phase (the period ranging from a week to several months post-injury) remains poorly understood. Initial damage and attendant responses dissipate over the acute phase, with evidence that glial activation and inflammatory processes persist into the subacute phase. Against this background, defensive responses to external challenges may be altered. Understanding the direction and course of defensive responses would add to predictability of the development of emotional sequelae in the aftermath of mTBI.

Replicating our earlier report (Pang et al., 2015) and consistent with the literature (Xing et al., 2013), exposure to mild LFP injury severely and persistently attenuated ASRs; attenuated ASRs were evident without remission 1 month after injury. Although ASRs were affected, CORT reactivity to a single foot shock did not differ in mTBI and SHAM rats. Peak CORT responses were at a level consistent with a moderately intense stressor; recovery did not differ between mTBI and SHAM rats. Previous reports have observed blunted CORT reactivity after 30 min restraint stress (Taylor et al., 2006) or forced swim (Taylor et al., 2008) in mTBI induced through CCI in the time frames post-injury studied herein. Suppressed CORT was also observed after restraint in mTBI induced through LFP (Griesbach et al., 2011). Inasmuch as foot shock is brief and discrete, the suppressed CORT responses observed by the Taylor group may reflect differences in more sustained adaptation to stressors and feedback, as opposed to the immediate response and its recovery.

Relatively novel endpoints for mTBI and stress are neurosteroids. There is a growing case that neurosteroids influence the trajectory of recovery after mTBI (Djebaili et al., 2005; Vanlandingham et al., 2007; Kilts et al., 2010; Marx et al., 2016; Melcangi et al., 2016). PREG (Del Cerro et al., 1996; Garcia-Estrada et al., 1999) and ALLO (Djebaili et al., 2004, 2005; Kelley et al., 2008) are neuroprotective and ALLO and ANDRO potent positive allosteric GABA_A_ receptor modulators (Wilson and Biscardi, 1997) that have anxiolytic and antidepressant effects (Marx et al., 2006a; Girdler and Klatzkin, 2007; Schule et al., 2011, 2014; Ben Dor et al., 2015). Treatment with PREG affected connectivity of neurocircuitry related to anxiety and depression (Sripada et al., 2014). Together, these observations suggest that poor outcomes in the aftermath of mTBI are related to low levels or reduced reactivity of neurosteroids.

However, across serum and all brain tissues measured control levels of ALLO, PREG, or ANDRO were not persistently affected by mTBI. Basal neurosteroids have been shown to be acutely elevated in the aftermath of neurotrauma (Meffre et al., 2007). It should be noted that the levels of control rats are not basal, but reflect activation from the experience of transfer and exposure to the experimental chamber without shock. Indeed these levels are consistent with previous work with acute mild stress (Paul and Purdy, 1992; Vallee et al., 2000). Although not basal, control neurosteroids levels provided a basis for understanding the degree of activation from the experience of an acute foot shock.

Exposure to acute stress in the form of single shock, a single shock after pre-exposure to the shock context, or two shocks separated by a day did not consistently or robustly affect neurosteroids in the serum. Modest, albeit inconsistent, elevations were apparent in ANDRO, to a lesser extent ALLO, with serum PREG the least responsive. A high degree of variability among serum samples decreased sensitivity to stress activation. Further, mTBI did not appreciably affect stress-induced serum neurosteroids.

In contrast to serum, distinct patterns of brain activation of neurosteroids were apparent. For PREG, stress induced elevations were evident in the prefrontal cortex and hippocampus. In the hippocampus, exposure to a single shock resulted in elevated PREG levels which adapted as levels after two shocks were no different than context controls. mTBI did not modify stress-induced PREG elevations. For ALLO, no distinction patterns of activation were apparent from stress or mTBI. The most stress-responsive neurosteroid under these conditions was ANDRO. Brain levels of ANDRO exhibited graded stress-induced patterns. In the prefrontal cortex, both mTBI and SHAM rats exhibited stepwise increases in ANDRO levels related to shock. Stress-induced increases were also apparent in the hippocampus and cerebellum, but not the degree of prefrontal cortex ANDRO. Furthermore, a coherent pattern of activation was apparent in mTBI rats. Overall, elevated ANDRO levels were apparent in mTBI compared to SHAMs in the prefrontal cortex and hippocampus; the mTBI-induced elevations were attributable to shock-stressed mTBI rats. Thus, brain ANDRO exhibited two interesting features during the subacute phase in the aftermath of mTBI: stress-sensitivity and further stress-induced activation in those experiencing mTBI.

Enhanced ANDRO activation in stressed mTBI rats has interesting implications. Given actions of ANDRO at GABA_A_ receptors, and the roles of the prefrontal cortex and hippocampus in affective processing, one may expect mTBI rats to be less anxious and better able to cope with stress. However, clinical observations are to the contrary with veterans exposed to blast mTBI exhibiting decreased levels of PREG and ANDRO (Marx et al., 2016), and active duty military and veterans experiencing mTBI exhibiting high rates of depression and suicidality (Bryan et al., 2014, 2015). Deficient ANDRO levels and reactivity in mTBI may only become manifest with sustained or chronic stressors. The apparent sensitivity of brain ANDRO levels to discrete acute stressors warrants future research in terms of stressor intensity and duration and the ability to cope with stress.

In sum, rats exposed to LFP injury consistent with mTBI exhibit severely and persistently attenuated ASRs. The deficiency in behavioral defensive responses did not extend to physiological endpoints. Clearly, CORT reactivity to a discrete acute stressor was unaffected. Across the neurosteroids assessed in serum and selected brain areas, neurosteroid reactivity was either normal or enhanced in mTBI induced by LFP in rats. Thus, behavioral and physiological defensive reactions appear to be divergent during the subacute period following mTBI in rats. The enhanced stress reactivity of brain ANDRO levels may provide resiliency to offset the deficiency in behavioral defensive responding in mTBI rats.

## Author contributions

RS, CM, SS, PA, and KP designed the study. CM, JK, and JN assayed and interpretted neurosteroids. RS, SS, PA, and KP analyzed behavioral and physiological data. RS performed the statistical analysis and wrote the manuscript. All edited.

### Conflict of interest statement

The authors declare that the research was conducted in the absence of any commercial or financial relationships that could be construed as a potential conflict of interest.
